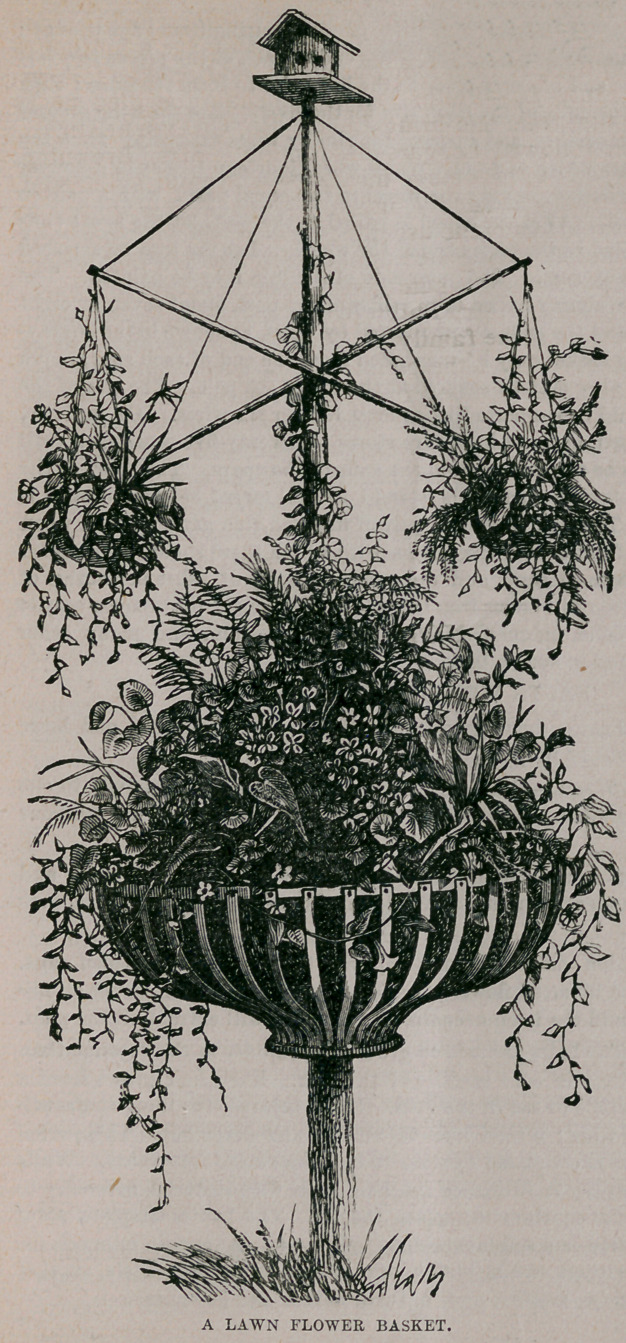# Household

**Published:** 1888-08

**Authors:** 


### HOUSEHOLD.

A LAWN FLOWER BASKET.

A LAWN FLOWER BASKET.—The foregoing is a design for a very pretty lawn flower stand or basket, which can be easily and cheaply constructed out of materials that are everywhere accessible. The center pole may be twenty feet high, if desirable. It should be set at least three feet into the ground, and for oue foot below the surface of the earth the pole should be smeared with a generous coat of tar. In the country an ornamental tree may be employed in lieu of the pole. The basket is made of hoop iron one inch wide ; ninety feet of it will be required, and forty rivets, such as are employed for uniting the ends of hoops for tubs and pails. such rivets can always be obtained at hardware stores. Let a portion of the hoop iron be cut into thirty-six pieces, two feet long, for the sides, and one piece twelve feet and three inches in length for the rim. Now punch thirty-six holes four inches apart, exactly in the middle of the long piece, measuring from edge to edge; after which punch a hole half an inch from the end of each of the pieces two feet in length, and rivet one end of every piece to the hoop rim to form the sides; then punch two holes near the ends of the hoop rim for the rivets, place the hoop around the post, rivet the ends together, and suspend the rim by wires extending from the rim to the pole. After which saw out a plank wheel fourteen inches in diameter for the bottom ef the basket, having the wheel in two equal parts, with a gain in each part to fit the post. After the wheel is nailed to the post, let the lower ends of the side irons be nailed to the periphery of the wheel. Let the iron be smeared with coal tar to prevent rust. The outer side of the iron may be painted. The two horizontal spars are simply nailed to the sides of the post, about seven or eight feet from the ground, and the ends are supported by wires, as shown in the engraving. A small hanging basket may be suspended from the end of each spar, and a neat little bird-house also may be secured at the top of the pole. The rim of the large basket post should be supported by at least four or six wires. Ox muzzles, which can be purchased at most hardware stores for twenty-five cents each, will make pretty baskets to be suspended from the ends of the spars. That part of the pole which the large basket surrounds should be well tarred before the earth is placed in the basket. The soil should consist of fine and rich garden mold, when one cannot procure leaf mold from the woods, with which there should be mingled say three or four quarts of sand and the same quantity of ashes per bushel of mold, with a quart of iron fillings or iron turnings. Verbenas, portulacca, scarlet geranium, abronia, lobelia, mignonette, loasa, sweet alyssum, and many other flowers may be cultivated in such a basket.

### FACTS WORTH KNOWING.

Oil of cinnamon will cause the disappearance of warts, however hard and large they may be. There will be no pain.

A Valuable Toilet Wash.—Ten cents' worth of gum benzoin dissolved in one pint of alcohol. Use a tablespoonful of this in the water which you use for your face.

To remove acid stains from linen or cotton goods, wet the cloth with water and hold a lighted match under the stain. The sulphurous gas from the match removes the stain.

The pain from a felon can be at once relieved by smoke from woolen rags. Place the rags under an inverted flower pot and put coals upon them, or set on fire some other way, then hold the felon over the smoke and it will extract all the pain.

Growth of Hair.—The hairs are said to grow more rapidly in the daytime than at night and in warm than in cold seasons of the year. Each hair grows from a bulb contained in a follicle or sac in the skin. If not injured, the limit of growth is reached at a certain time; it then loses its vitality and drops out. This period of life in robust persons is estimated by some to be from two to four years. While the follicle remains healthy it furnishes the hair bulb with material necessary to its growth, and new hairs continue to replace the old. The hair is simply a plant with a bulbous root placed in a soil favorable to its growth. At certain intervals the stalk ceases growing, withers and is broken off. From the same follicle another shoot is sent forth to grow, mature, and, in turn, wither and disappear.

## Figures and Tables

**Figure f1:**